# Long-term impact of baseline serum uric acid levels on living kidney donors: a retrospective study

**DOI:** 10.1186/s12882-021-02295-0

**Published:** 2021-03-12

**Authors:** Kosuke Tanaka, Shigeyoshi Yamanaga, Yuji Hidaka, Sho Nishida, Kohei Kinoshita, Akari Kaba, Toshinori Ishizuka, Satoshi Hamanoue, Kenji Okumura, Chiaki Kawabata, Mariko Toyoda, Akira Miyata, Masayuki Kashima, Hiroshi Yokomizo

**Affiliations:** 1 Department of Surgery, Japanese Red Cross Kumamoto Hospital, 861-8520, 2-1-1 Nagamine Minami, Higashi-ku, Kumamoto, Japan; 2 Department of Nephrology, Japanese Red Cross Kumamoto Hospital, Kumamoto, Japan

**Keywords:** Serum uric acid, Living kidney donors, Adverse events

## Abstract

**Background:**

Preoperative characteristics of living kidney donors are commonly considered during donor selection and postoperative follow-up. However, the impact of preoperative uric acid (UA) levels is poorly documented. The aim of this study was to evaluate the association between preoperative serum UA levels and post-donation long-term events and renal function.

**Methods:**

This was a single-center retrospective analysis of 183 living kidney donors. The donors were divided into high (≥5.5 mg/dl) and low (< 5.5 mg/dl) UA groups. We analyzed the relationship between preoperative UA levels and postoperative estimated glomerular filtration rate (eGFR), as well as adverse events (cardiovascular events and additional prescriptions for hypertension, gout, dyslipidemia, and diabetes mellitus), over 5 years after donation.

**Results:**

In total, 44 donors experienced 52 adverse events over 5 years. The incidence of adverse events within 5 years was significantly higher in the high UA group than in the low UA group (50% vs. 24%, *p* = 0.003); this was true even after the exclusion of hyperuricemia-related events (*p* = 0.047). UA emerged as an independent risk factor for adverse events (*p* = 0.012). Donors with higher UA levels had lower eGFRs after donation, whereas body mass index, hemoglobin A1c, blood pressure, and low-density lipoprotein cholesterol did not have any impact on the eGFR.

**Conclusions:**

The findings suggest that preoperative UA levels should be considered during donor selection and postoperative follow-up.

## Introduction

Living donor kidney transplantation is one of the established treatment options for patients with end-stage renal diseases (ESRDs) [1, 2]. Given the projected lifetime risk of ESRD in living donors, [3–6] their safety is of utmost importance, as highlighted in the existing guidelines [7, 8].

Uric acid (UA) is known to induce endothelial dysfunction by reactive oxygen production, [9] inflammation and vasoconstriction by various mechanisms, [10, 11] and increased cyclooxygenase-2 expression [12]. Through these mechanisms, hyperuricemia exacerbates vascular disorders, [13–16] renal arteriolar damage, [17, 18] and glomerulosclerosis [10]. Furthermore, recent clinical studies revealed that hyperuricemia was an independent risk factor for cardiovascular events and the progression of chronic kidney diseases (CKD) in the general population, [19–22] which is also true for patients with CKD and kidney transplant recipients [23, 24].

The impact of serum UA levels on the residual kidney of living kidney donors has been an emerging topic of interest. A large cohort study including 4650 living donors showed that donors with post-donation gout had a higher risk of acute kidney failure and progression of CKD [25]. Other studies from Korea and Turkey suggested that preoperative hyperuricemia predicted impaired postoperative renal functions at 6 and 12 months in living donors [26–28]. We have also reported that preoperative hyperuricemia was strongly associated with suboptimal renal compensatory hypertrophy at 12 months after renal donation [29].

Although no statement regarding hyperuricemia was included in the Amsterdam Forum on the Care of the Live Kidney Donor published in 2005, [7] given these emerging studies on hyperuricemia for the general population and living donors, the 2017 Kidney Disease Improving Global Outcomes Clinical Practice Guideline on the Evaluation and Care of Living Kidney Donors referred to the potential impact of UA levels on living donors [8]. However, the prognostic significance of hyperuricemia in living donors remains scarce so far. Therefore, in the present study, we hypothesized that preoperative serum UA levels would be associated with post-donation long-term events and renal function.

## Patients and methods

### Study population

This was a single-center, retrospective study. Between May 2011 and January 2020, a total of 187 living kidney transplantations were performed at the Japanese Red Cross Kumamoto Hospital. Four cases were excluded due to the unavailability of postoperative data. Finally, 183 patients were included in the study. The follow-up rate at 5 years was 92.0%. Preoperative/postoperative clinical factors, postoperative adverse events, and the estimated glomerular filtration rate (eGFR) were analyzed. The main endpoint was the occurrence of postoperative adverse events. Postoperative adverse events were defined as the occurrence of clinical events including cardiovascular events, induction of dialysis, and de novo prescriptions for hypertension, hyperuricemia, dyslipidemia, and diabetes mellitus. Donors were divided into two groups based on their preoperative UA levels: the high UA group (*n* = 57, UA level ≥ 5.5 mg/dl) and the low UA group (*n* = 126, UA level < 5.5 mg/dl). The median value of UA in the present study was 4.9 [4.2–5.7] mg/dl. The definitive cutoff value of serum UA level as the risk factor of metabolic syndrome or CKD was still controversial [30–36]. The cut-off value of 5.5 mg/dl was approximately the 75th percentile of the distribution of all donors included in this study.

All donors were selected with strict compliance with the Japanese donor selection criteria, [37] which includes details about age (≤80), blood pressure (BP) (< 140/90 mmHg or ≤ 130/80 with antihypertensive medication), body mass index (BMI) (≤32 kg/m^2^), GFR (≥70 ml/min/1.73 m^2^), hemoglobin A1c (HbA1c) (≤6.5%), and no systemic or infectious disease. This investigation conformed with the principles outlined in the Declaration of Helsinki of 1964 and the Declaration of Istanbul of 2018. The study was approved by the Institutional Review Board of the Japanese Red Cross Kumamoto Hospital (study approval number 432). The review board waived the requirement for informed consent for this research. None of the transplant donors were from a vulnerable population and all donors or next of kin provided freely given written informed consent.

### Recording and assessment of clinical data

We retrospectively collected all clinical data including the serum UA levels from the medical records. Most of the donors’ baseline BP readings were collected from 24-h BP monitoring, and the average values of the systolic BP recorded in the afternoon were adopted. HbA1c data collected at the time of initial drawing (mainly three months before donor nephrectomy) were included. In 21 cases, HbA1c was measured using the Japan Diabetes Society (JDS) methods, which were used until April 2012. Since then, HbA1c has been measured by the National Glycohemoglobin Standardization Program (NGSP) method. The HbA1c values measured by the JDS methods were converted into NGSP HbA1c values using the following formula [38]: NGSP value (%) = 1.02 × JDS value (%) + 0.25%. eGFR was calculated using the formula recommended by the Japanese Society of Nephrology [39]: eGFR (ml/min/1.73 m^2^) = 194 × creatinine (Cr)^-1.094^ × Age^-0.28^ in male donors and 194 × Cr^-1.094^ × Age^-0.28^ × 0.739 in female donors. Creatinine clearance (CCr) was measured twice, and the average value was adopted.

### Statistical analysis

All data were analyzed using SPSS (version 25, IBM Corp., Armonk, NY, USA). Age, height, weight, BMI, BP, HbA1c, UA, low-density lipoprotein cholesterol (LDL-C), CCr, and eGFR were regarded as continuous data. These data are expressed as medians and interquartile ranges and were compared between two groups using the Mann-Whitney U test as the sample size of the study was small. The Chi-squared (χ^2^) test was used for the analysis of categorical data such as sex and tobacco use. The hazard ratio (HR) for adverse events was calculated by adjusting for preoperative factors including sex, age, BMI, BP, HbA1c, UA, and LDL-C. The cumulative incidence rate of postoperative adverse events was analyzed using the Kaplan-Meier method. Statistical significance was calculated using the log-rank test for two groups and Cox regression analysis for more segmentalized UA groups. The optimal cut-off level of preoperative UA levels was analyzed with the help of the receiver operating characteristic (ROC) curve. The impact of BMI, HbA1c, BP, and LDL-C on postoperative adverse events and eGFR was also analyzed. A post hoc power calculation was performed to detect the statistical significance of the UA levels. Post hoc Power Calculator (https://clincalc.com/stats/Power.aspx) was used for the power calculation.

There were no missing data apart from those related to tobacco use. There were missing data regarding tobacco use in 32 patients; thus, tobacco use was not included in the further multivariable analysis. A *p-*value < 0.05 was considered statistically significant. The Strengthening the Reporting of Observational Studies in Epidemiology (STROBE) statement guidelines for reporting of observational studies has been followed in this study [40].

## Results

### Donor characteristics

The donors’ characteristics are shown in Table 1. The median age of all donors was 58 years. Sixty-five donors (35.3%) were male. UA levels were 6.4 [5.7–6.9] mg/dl in the high UA group and 4.5 [3.7–4.9] mg/dl in the low UA group (*p* < 0.001). The proportion of male donors (high UA: 68.4% vs. low UA: 20.6%, *p* < 0.001), values of BMI (high UA: 24.6 kg/m^2^ [21.9–27.0] vs. low UA: 22.1 kg/m^2^ [20.7–24.2], *p* < 0.001), and BP (high UA: 129 [119–142] mmHg vs. low UA: 122 [111–132], *p* = 0.003) were significantly different between the two groups. Preoperative eGFR was not significantly different between the two groups.

**Table 1:** Baseline characteristics of living donors

	All donors (*n* = 183)	High UA group (*n* = 57)	Low UA group (*n* = 126)	*p-*value
Sex: male (%)	65 (35.3)	39 (68.4)	26 (20.6)	< 0.001
Age, years	58 [51–65]	58 [52–63]	58 [49–65]	0.963
Tobacco use, *n* (%)				0.179
Non-smoker	102 (55.7)	27 (47.4)	75 (59.5)	
Current smoker	32 (17.5)	14 (24.6)	18 (14.3)	
Ex-smoker	17 (9.3)	5 (8.8)	12 (9.5)	
Height, cm	159 [154–166]	167 [160–173]	157 [153–161]	< 0.001
Weight, kg	59.0 [52.0–66.1]	65.0 [59.5–74.7]	54.1 [50.0–62.9]	< 0.001
BMI, kg/m^2^	22.9 [20.9–25.1]	24.6 [21.9–27.0]	22.1 [20.7–24.2]	< 0.001
Systolic BP, mmHg	124 [115–136]	129 [119–142]	122 [111–132]	0.003
HbA1c, %	5.6 [5.4–5.9]	5.6 [5.4–5.9]	5.6 [5.5–5.9]	0.59
UA, mg/dl	4.9 [4.2–5.7]	6.4 [5.7–6.9]	4.5 [3.7–4.9]	< 0.001
LDL-C, mg/dl	120 [102–138]	120 [106–144]	118 [100–137]	0.814
CCr, ml/min	106.5 [95.3–121.4]	105.4 [95.9–119.0]	107.8 [95.0–122.9]	0.39
preoperative eGFR, ml/min/1.73 m^2^	81.1 [73.0–91.8]	76.5 [72.2–87.0]	82.9 [74.1–92.7]	0.064

Values are expressed as median [interquartile range] otherwise noted. *UA,* uric acid; *BMI* body mass index; *BP,* blood pressure; *HbA1c,* hemoglobin A1c; *LDL-C,* low-density lipoprotein cholesterol; *CCr,* creatinine clearance; *eGFR*, estimated glomerular filtration

### Changes in preoperative and postoperative UA levels

A significant increase in UA levels (+ 0.9 [0.4–1.5] mg/dl, *p* < 0.001) was observed from preoperative (4.9 [4.2–5.7] mg/dl) to 1-year postoperative (5.8 [5.0–6.8] mg/dl) in all donors. The degree of increment was similar in male (+ 1.0 [0.3–1.6] mg/dl from 5.9 [5.0–6.7] mg/dl to 6.8 [6.0–7.9] mg/dl, *p* < 0.001) and female (+ 0.9 [0.4–1.4] mg/dl from 4.5 [3.8–5.1] mg/dl to 5.4 [4.6–6.3] mg/dl, *p* < 0.001) donors. In addition, the degree of increment was similar in the high UA (+ 0.9 [0.1–1.4] mg/dl from 6.4 [5.7–6.9] mg/dl to 7.2 [6.6–8.2] mg/dl, *p* < 0.001) and low UA groups (+ 0.9 [0.4–1.5] mg/dl from 4.5 [3.7–4.9] mg/dl to 5.4 [4.5–6.2] mg/dl, p < 0.001). The annual value of postoperative UA levels were 5.8 [4.8–6.9] mg/dl at 2 years, 6.1 [4.9–6.9] mg/dl at 3 years, 6.1 [4.9–6.9] mg/dl at 4 years, and 6.2 [5.0–6.7] mg/dl at 5 years after donation.

### Adverse events

A total of 44 (24%) donors experienced 52 adverse events in the 5 years after donation. Eight cases experienced more than two adverse events. The observed adverse events were as follows: two cases with myocardial infarction, one with cerebral infarction, one with the induction of hemodialysis due to de novo IgA nephropathy, and 48 with de novo prescriptions (eight associated with hyperuricemia, 14 with hypertension, 23 with dyslipidemia, and three with hyperglycemia). Xanthine oxidase inhibitors were prescribed for all cases with hyperuricemia. Calcium blockers and angiotensin-converting enzyme inhibitors were prescribed for eight and six cases with hypertension, respectively. In dyslipidemia, statins were prescribed for 21 cases, whereas eicosatetraenoic acid and intestinal cholesterol transporter inhibitor were prescribed for one case each. Biguanide antihyperglycemic agent and dipeptidyl peptidase-4 inhibitors were prescribed for one and two cases of hyperglycemia, respectively. There was no mortality in this cohort.

### Cumulative incidence rate of adverse events

**Fig. 1:**
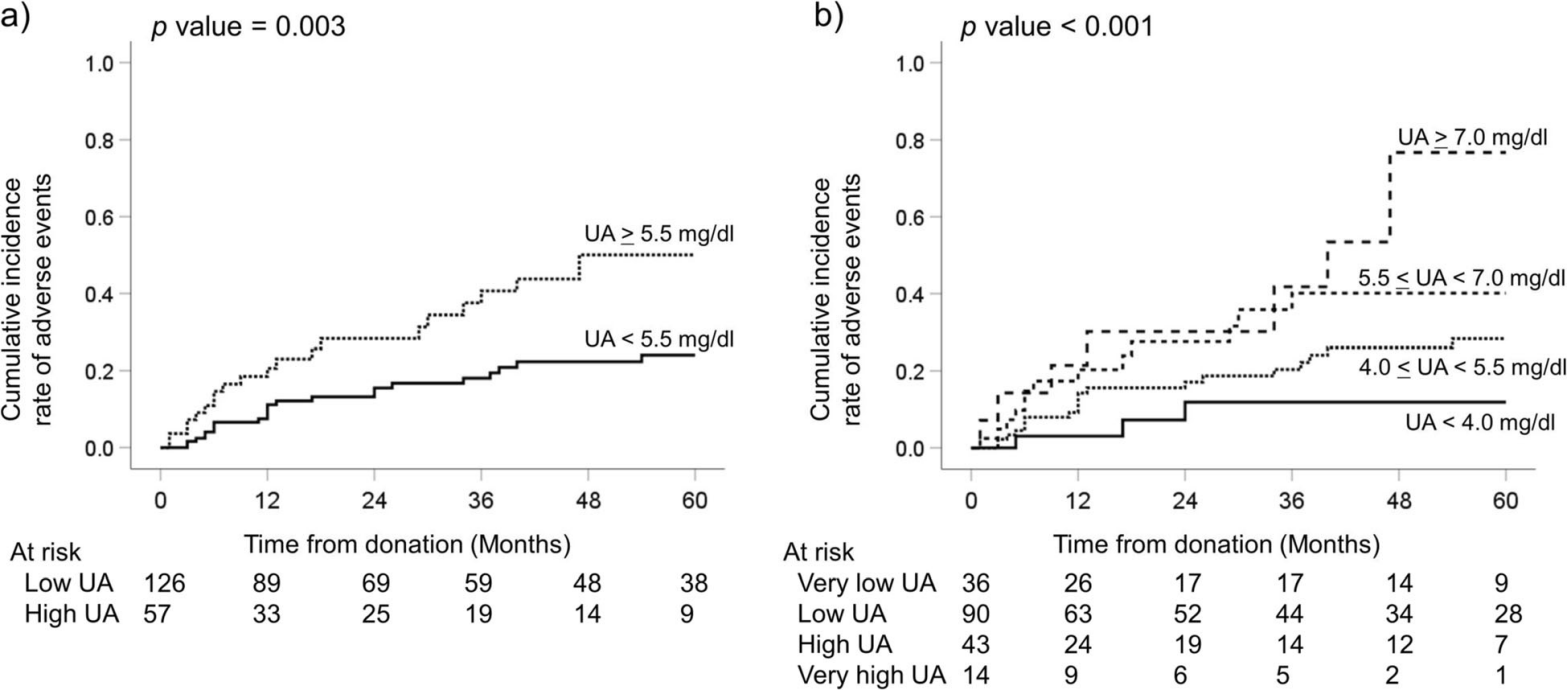
The cumulative incidence rate of adverse events for **a**) the high vs low UA groups and **b**) granular analysis of UA levels divided by 1.5 mg/dl. *UA* uric acid

**Fig. 2:**
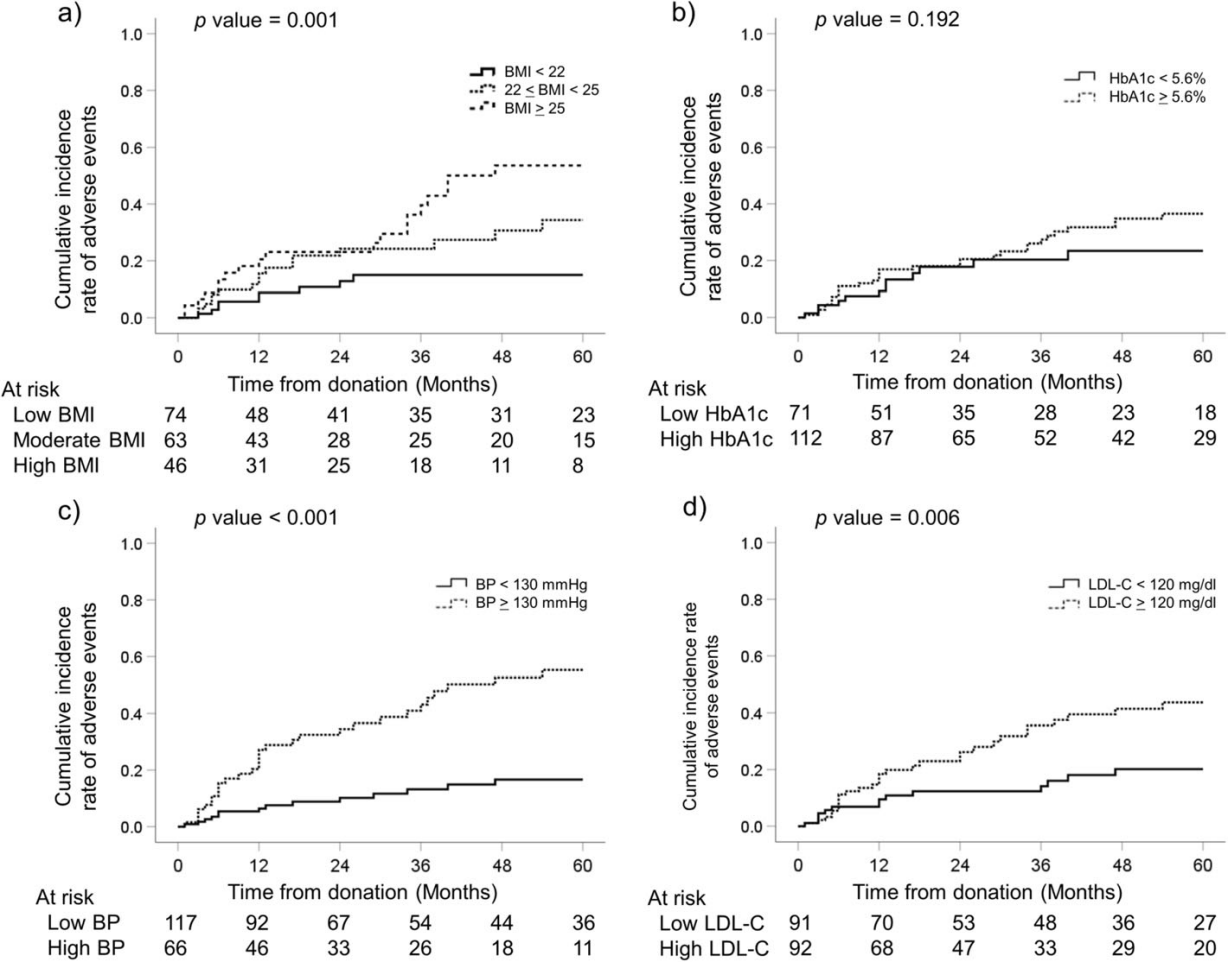
The analysis of adverse events for **a**) BMI, **b**) HbA1c, **c**) BP, and **d**) LDL-C. *BMI,* body mass index; *BP,* blood pressure; *HbA1c,* hemoglobin A1c; *LDL-C,* low-density lipoprotein cholesterol

Fig. 1 shows the analysis of the cumulative incidence of post-donation adverse events. The high UA group showed a significantly higher incidence of adverse events within 5 years after donation than the low UA group (50% vs. 24%, *p* = 0.003, Fig. 1a). The granular analysis of adverse events (cutoff UA ± 1.5 mg/dl) also showed a significant gradual increment in the incidence with each UA level (*p* < 0.001, Fig. 1b). A considerable proportion of donors (77%) with very high preoperative UA levels (≥7.0 mg/dl) experienced adverse events within 5 years after donation. Higher BMI, BP, and LDL-C were also significantly different between the two groups, whereas no significant difference was found for HbA1c (Fig. 2). The cumulative incidence of adverse events, except for the prescription for hyperuricemia, was also significantly different between the two groups (*p* = 0.047, log-rank test).

In the univariable analyses, preoperative UA levels (HR [per 1 mg/dl]: 1.643, 95% confidence interval [CI]: 1.248–2.163, *p* < 0.001), BMI (HR [per 1 kg/m^2^]: 1.278, 95% CI: 1.131–1.445, *p* < 0.001), BP (HR [per 10 mmHg]: 1.884, 95% CI: 1.447–2.454, *p* < 0.001), and LDL-C (HR [per 10 mg/dl]: 1.272, 95% CI: 1.127–1.435, *p* < 0.001) were significantly associated with a higher incidence of adverse events in the 5 years after donation (Table 2). After the adjustment, UA (adjusted HR [per 1 mg/dl]: 1.759, 95% CI: 1.130–2.737, *p* = 0.012), BP (adjusted HR [per 10 mmHg]: 1.895 95% CI: 1.395–2.574, *p* < 0.001), and LDL-C (adjusted HR [per 10 mg/dl]: 1.357, 95% CI: 1.164–1.582, *p* < 0.001) levels emerged as independent risk factors for the development of adverse events. The ROC curve revealed that the cut-off value of preoperative UA levels was 5.5 mg/dl but the area under the curve was 0.659, which was not reliable. Table 3 showed the baseline characteristics of donors regarding the presence of adverse events. Weight, BMI, systolic BP, UA, and LDL-C were significantly different between the two groups.

**Table 2:** Cox regression analysis for adverse events

	Univariable analysis		Multivariable analysis	
	HR [95% CI]	*p-*value	aHR [95% CI]	*p-* value
Sex (ref. female)	0.738 [0.368–1.480]	0.392	1.387 [0.465–4.141]	0.557
Age (years, per 10)	1.145 [0.808–1.622]	0.447	1.147 [0.671–1.960]	0.617
BMI (kg/m^2^, per 1)	1.278 [1.131–1.445]	< 0.001	1.124 [0.963–1.312]	0.140
BP (mmHg, per 10)	1.884 [1.447–2.454]	< 0.001	1.895 [1.395–2.574]	< 0.001
HbA1c (%, per 0.1)	1.056 [0.961–1.160]	0.256	0.994 [0.875–1.130]	0.930
UA (mg/dl, per 1)	1.643 [1.248–2.163]	< 0.001	1.759 [1.130–2.737]	0.012
LDL-C (mg/dl, per 10)	1.272 [1.127–1.435]	< 0.001	1.357 [1.164–1.582]	< 0.001
preoperative eGFR (ml/min/1.73m^2^, per 1)	0.997 [0.974–1.021]	0.824	1.032 [0.997–1.068]	0.075

*BMI,* body mass index*; BP,* blood pressure; *HbA1c,* hemoglobin A1c; *UA,* uric acid; *LDL-C,* low-density lipoprotein cholesterol; *eGFR*, estimated glomerular filtration rate; *HR,* hazard ratio; *aHR,* adjusted hazard ratio; *CI* confidence interval

**Table 3:** Baseline characteristics of living donors regarding adverse events

	All donors (*n* = 183)	Donors without adverse events(*n* = 139)	Donors with adverse events(*n* = 44)	*p* value
Sex: male (%)	65 (35.3)	47 (33.8)	18 (40.9)	0.391
Age (years)	58 [51–65]	58 [50–65]	59 [53–65]	0.497
Tobacco use, *n* (%)				0.07
Non-smoker	102 (55.7)	80 (57.6)	22 (50.0)	
Current smoker	32 (17.5)	21 (15.1)	11 (25.0)	
Ex-smoker	17 (9.3)	16 (11.5)	1 (2.3)	
Height (cm)	159 [154–166]	159 [154–166]	160 [154–166]	0.467
Weight (kg)	59.0 [52.0–66.1]	57.0 [50.9–65.0]	64.6 [55.3–74.2]	< 0.001
BMI (kg/m^2^)	22.9 [20.9–25.1]	22.1 [20.7–24.7]	24.4 [22.6–27.4]	< 0.001
BP (mmHg)	124 [115–136]	122 [112–130]	137 [125–147]	< 0.001
HbA1c (%)	5.6 [5.4–5.9]	5.6 [5.4–5.9]	5.7 [5.5–6.0]	0.153
UA (mg/dl)	4.9 [4.2–5.7]	4.8 [4.0–5.5]	5.2 [4.5–6.7]	0.001
LDL (mg/dl)	120 [102–138]	117 [99–132]	135 [115–164]	< 0.001
CCr (ml/min)	106.5 [95.3–121.4]	107.3 [95.3–122.5]	106.0 [93.5–120.7]	0.712
preoperative eGFR (ml/min/1.73 m2)	81.1 [73.0–91.8]	81.1 [72.7–92.2]	82.1 [74.9–89.2]	0.971

median (IQR). *UA* uric acid. *BMI* body mass index. *BP* blood pressure. *HbA1c* hemoglobin A1c. *LDL* low-density lipoprotein. *CCr* creatinine clearance. *eGFR* estimated glomerular filtration

### Transition of postoperative eGFR

**Fig. 3:**
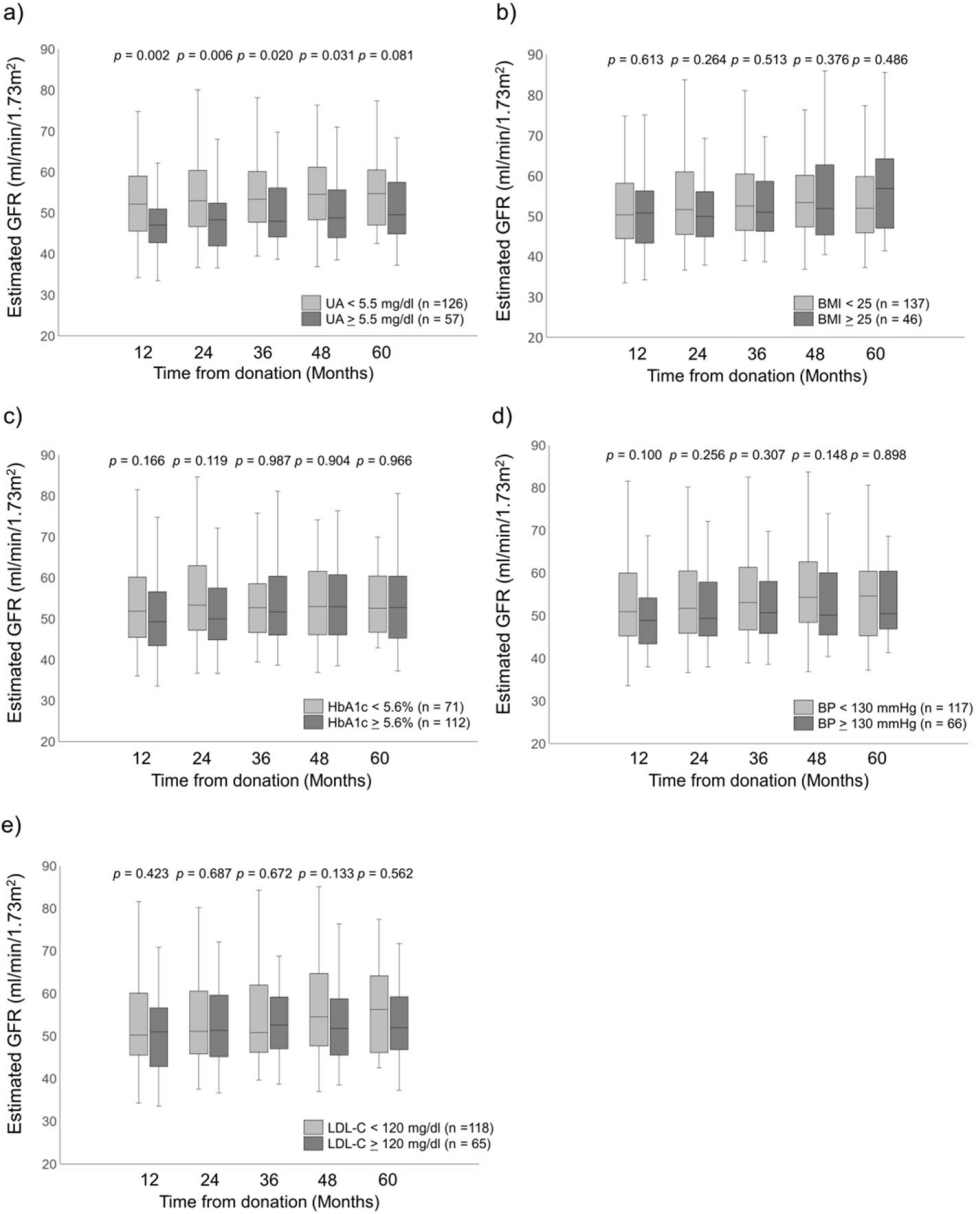
Postoperative eGFR of **a**) UA, **b**) BMI, **c**) HbA1c, **d**) BP, and **e**) LDL-C. *UA,* uric acid; *BMI,* body mass index; *BP,* blood pressure; *HbA1c,* hemoglobin A1c; *LDL-C,* low-density lipoprotein cholesterol; *eGFR,* estimated glomerular filtration

Fig. 3 demonstrates the postoperative transition of eGFR. The high UA group showed significantly lower postoperative eGFR than the low UA group from 1 to 4 years after donation (high vs. low UA group; 1 year: 47.0 ml/min/1.73 m^2^ [42.7–50.9] vs. 52.2 ml/min/1.73 m^2^ [45.6–59.0], *p* = 0.002; 2 years: 48.3 ml/min/1.73 m^2^ [41.9–52.4] vs. 53.0 ml/min/1.73 m^2^ [46.6–60.4], *p* = 0.006; 3 years: 48.0 ml/min/1.73 m^2^ [44.1–56.1] vs. 53.3 ml/min/1.73 m^2^ [47.7–60.1], *p* = 0.020; 4 years: 48.8 ml/min/1.73 m^2^ [44.0–55.6] vs. 54.5 ml/min/1.73 m^2^ [48.3–61.2], *p* = 0.031; 5 years: 49.5 ml/min/1.73 m^2^ [44.9–57.5] vs. 54.7 ml/min/1.73 m^2^ [47.0–60.5], *p* = 0.081).

### Post-hoc power calculation

A post-hoc power calculation was conducted to detect the significance of UA levels. The incidence rate in the two groups was used for the dichotomous endpoint, and the median and interquartile range for the continuous endpoint. An alpha error rate of 5% was set. This calculation revealed sufficient power of the effects of UA on postoperative adverse events (93.2%) and the 1-year eGFR (90.7%).

## Discussion

In the present study, we showed that higher UA levels may be associated with post-donation adverse events and negative effects on eGFR in living donors. There are only a few short-term studies available that discuss the correlation between preoperative UA levels and postoperative renal function [26–28]. Cho et al. [27] concluded that a 1 mg/dl increase in the preoperative UA level was associated with a 1.7-fold higher risk of eGFR decline (> 25%) at 6 months after donation in females. However, this trend was not observed in males. Similarly, Bravo et al. [28] demonstrated the association of high UA levels (> 4.5 mg/dl) with the decrease in eGFR at 6 months and 1 year after donation in females only. However, Kulah [26] showed that these associations were also applicable to males. This author found that UA levels higher than 6 mg/dl in males and 5 mg/dl in females were associated with high Cr levels (> 1.4 mg/dl in males and > 1.3 mg/dl in females) at 6 months post-surgery. The present study corroborated these prior studies and, furthermore, extended them by demonstrating the impact of baseline UA levels on adverse events related to the decline in renal function irrespective of sex.

Although higher UA had a negative impact on the postoperative eGFR recovery within the 4 years after donation in the present study, other factors such as BMI, BP, HbA1c, and LDL-C were found to be unrelated to the poor recovery of renal function. Nevertheless, hypertension, [41–43] hyperglycemia, [44, 45] and dyslipidemia [46, 47] have been reported to be associated with a decline in renal function in the general population. This discrepancy might be explained by the fact that a donor loses 50% of the renal function immediately after the nephrectomy. This sudden decline in renal function could affect UA excretion, which would certainly lead to higher UA levels [48, 49]. After living donation, UA levels increased by 15% (4.6 mg/dl to 5.3 mg/dl) at 6 months in a prior multi-center study [50] and by 20% (from 4.9 mg/dl to 5.8 mg/dl) at 1 year in the present study.

Based on the existing literature, we speculate that the sudden increase in UA after living donation might result in the poor recovery of renal function through endothelial dysfunction, [9] inflammation, vasoconstriction, [10, 11] and increased COX-2 expression [12], which may eventually lead to hypertension, [51, 52] hyperglycemia, and dyslipidemia [53, 54]. In fact, prior studies have suggested that hyperuricemia is associated with the occurrence of hypertension [51, 52] and dyslipidemia [53, 54] in the non-CKD population. The results of Kaplan-Meier analysis from a Turkish retrospective study revealed that post-donation new-onset hypertension was highly likely to occur in donors with high baseline uric acid level (> 4.5 mg/dL, *P* < 0.05) [55]. In the CKD population, a randomized control study revealed that a 1 mg/dL increase in the UA level was associated with a 17% increased risk of all-cause mortality (HR = 1.17) and a 16% increased risk of cardiovascular disease mortality (HR = 1.16) [56]. A meta-analysis suggested that a 1 mg/dl increase in UA level was correlated with the incidence of hypertension (relative risk  =  1.15) [57]. In addition, a Japanese cohort study showed that with every 1 mg/dL increase in the serum UA levels, there was a 19% increased risk for developing hypertension (odds ratio = 1.19, 95% CI: 1.11–1.27) [58]. The present study also revealed that the cumulative incidence of adverse events, except for the prescription for hyperuricemia, was significantly different between the high and low UA groups (*p* = 0.047, log-rank test). Although the population in the present study was different from the CKD population in previous studies, we have provided new insight into the possible impact of an immediate increase in UA levels on new-onset hypertension and hyperlipidemia after donation. According to a recent report, new-onset obesity might be related to preoperative UA level [59]. Further studies must be conducted to explore these findings in a larger patient population.

Similarly, in the CKD population, a sudden cessation of anti-hyperuricemia drugs (such as allopurinol) is known to induce renal and endothelial dysfunction [60]. Furthermore, the withdrawal of anti-hyperuricemia drugs in patients with mild hyperuricemia results in the worsening of hypertension and the acceleration of the rate of kidney function loss [52]. As noted above, living donors face a similar situation to patients with a sudden withdrawal of anti-hyperuricemia drugs through the sudden increase in the UA levels. Given the difference in the baseline characteristics between living donors and the CKD population, more studies are needed to find ways to mitigate the risk of adverse events for living donors after donation.

We set the arbitrary cutoff value for the UA level according to the distribution in the present study. This cutoff value (5.5 mg/dl) was slightly lower than the standard value for patients with asymptomatic hyperuricemia. While the Japanese Society of Gout and Nucleic Acid Metabolism has suggested that the cutoff value for serum UA be 7.0 mg/dl regardless of sex and age, [36] the American College of Rheumatologists and the American College of Physicians did not provide any recommendation regarding the cutoff and treatment [31, 32, 34, 35]. In recent years, asymptomatic hyperuricemia has also emerged as a target for the treatment of various conditions. The optimal UA levels associated with the lowest development of cardiometabolic diseases were suggested as < 5 mg/dL for men and 2–4 mg/dL for women [33]. Also, in an Italian cohort study, patients with mild hyperuricemia (4.6–5.5 mg/dl) had higher cardiovascular mortality (HR = 1.98, 95% CI: 1.22–3.23) than those with low UA levels (1.8–4.5 mg/dl) [30]. Given these emerging results, our cutoff of 5.5 mg/dl seemed reasonable. However, in our study, we did not demonstrate the reliable cut-off value with the help of a ROC curve. Further large randomized trials are warranted to elucidate the potential effect and treatment of asymptomatic hyperuricemia in living donors.

HbA1c was not associated with either adverse events or reduced eGFR. Normal HbA1c may not affect postoperative renal functions. A previous Japanese study at a single center reported that prediabetic donors without diabetic complications who showed an abnormal pattern in the 75 g oral glucose tolerance test did not develop ESRD after donation in the long-term [61]. Another study reported that although donors with impaired fasting glucose (100–125 mg/dL) could preserve their renal functions, a high proportion of them developed diabetes mellitus (15.56%) within 10 years after donation [62]. In our study, while 97.8% (179/183) of donors had non-diabetic HbA1c (< 6.5%), 61.2% (112/183) of the donors had prediabetic HbA1c (5.6–6.5%). The postoperative eGFR did not differ between the normal and prediabetic or diabetic HbA1c donors. Our study corroborated previous studies and suggested that, in addition to the abnormal fasting glucose, prediabetic HbA1c may also not be associated with postoperative renal functions at 5 years after donation.

Other clinical parameters, apart from HbA1c, were found to predict the postoperative incidence of adverse events in the present study. Higher BMI (> 22 or 25 kg/m^2^), higher BP (> 130 mmHg), and higher LDL-C levels (> 120 mg/dl) were associated with a greater occurrence of adverse events. These thresholds can be conceived as pre-disease status. The Japan Society for the Study of Obesity has defined obesity as a BMI of > 25 kg/m^2^ [63]. A Japanese cohort study demonstrated a reverse-J pattern for all-cause mortality, and the lowest risk of total mortality was observed in patients with a BMI of 21–27 kg/m^2^ [64]. The American Heart Association guidelines for hypertension (2017) has revised the definition of BP for hypertension by lowering the threshold from 140/90 mmHg to 130/80 mmHg [65]. A randomized trial showed that intensive systolic BP control (< 120 mmHg) resulted in lower all-cause mortality (HR = 0.73; 95% CI: 0.60–0.90) [66]. The Japan Atherosclerosis Society Guidelines for Prevention of Atherosclerotic Cardiovascular Diseases 2017 suggested that patients with a high risk of diabetes mellitus, CKD, non-cardiogenic cerebral infarction, or peripheral arterial disease should control LDL-C levels at < 120 mg/dl as the primary preventive measure for dyslipidemia [67]. These active interventions for pre-disease status have become more important in recent years. The present study showed that this pre-disease status had a negative impact on the incidence of lifestyle-related diseases after living donation, but not on the renal function itself.

Most of the donors (92.0%) were comprehensively followed up for 5 years in the present study. The Japanese Society for Clinical Renal Transplantation and the Japan Society for Transplantation reported in a follow-up survey in 2018 that 20.9% of Japanese living kidney donors’ data could be aggregated by web-based methods and 79.5% of them were properly followed up for 5 years after donation [68]. The United Network for Organ Sharing and Organ Procurement and Transplantation Network mandates a 2-year follow-up after donation [69]. The study that used data from the Scientific Registry of Transplant Recipients showed that only 43% of centers in the United States met the 6-month, 1-year, and 2-year living donor follow-up criteria [70]. An additional clinical study on the safety of American living donors reported that 85.3% of donors could not be properly assessed due to the cessation of follow-up or lack of data [71]. This report also showed a high possibility that non-surgical adverse events occurred more than 2 years after donation [71]. Given the results of these studies, both the follow-up rate and period of the present study were sufficient to show reliable results. Furthermore, the present study highlighted the importance of long-term follow-up for the safety of living kidney donors.

There are some limitations to this study. First, this was a single-center retrospective study with a relatively small sample size, and there were only 44 donors with 52 adverse events. This limited number of events may not be adequate to support meaningful multivariable analyses. In the present study, most of the living donors were limited to Japanese living in a single prefecture. Analysis of multiethnic and heterogeneous populations should be considered in further studies. Second, although de novo prescriptions are regarded as hard outcomes, both the timing and indication of the prescriptions are dependent on individual doctors. Living donors generally hesitate to take a new medication because they think they do not require it. Therefore, it is likely that the incidence rate in the present study was underestimated.

## Conclusion

In conclusion, we showed that preoperative UA levels may be one of the negative factors affecting the incidence of lifestyle-related adverse events and renal function after living kidney donation. Preoperative uric acid level should be considered when selecting living kidney donors. Further studies are warranted for mitigating the risk of morbidity and mortality in living donors.

## Data Availability

The datasets used during the present study are available from the corresponding author on reasonable request.

## Abbreviations

BMI: Body mass index
BP: Blood pressure
Cr: Creatinine
CCr: Creatinine clearance
CI: Confidence interval
CKD: Chronic kidney disease
eGFR: Estimated glomerular filtration rate
ESRD: End-stage renal disease
HbA1c: Hemoglobin A1c
HR: Hazard ratio
JDS: Japan Diabetes Society
LDL-C: Low-density lipoprotein cholesterol
NGSP: National Glycohemoglobin Standardization Program
ROC: Receiver operating characteristic
UA: Uric acid
